# Tolerance of Anaerobic Bacteria to Chlorinated Solvents

**DOI:** 10.1264/jsme2.ME13113

**Published:** 2014-01-17

**Authors:** Joanna C. Koenig, Kathrin D. Groissmeier, Mike J. Manefield

**Affiliations:** 1 Centre for Marine Bioinnovation, School of Biotechnology and Biomolecular Sciences, University of New South Wales, Sydney, Australia; 2 Helmholtz Institute of Groundwater Ecology, Ingolstaedter Landstrasse 1, D-85764, Neuherberg, Germany

**Keywords:** solvent tolerance, chlorinated solvents, anaerobic bacteria, toxicity

## Abstract

The aim of this research was to evaluate the effects of four chlorinated aliphatic hydrocarbons (CAHs), perchloroethene (PCE), carbon tetrachloride (CT), chloroform (CF) and 1,2-dichloroethane (1,2-DCA), on the growth of eight anaerobic bacteria: four fermentative species (*Escherichia coli*, *Klebsiella* sp., *Clostridium* sp. and *Paenibacillus* sp.) and four respiring species (*Pseudomonas aeruginosa*, *Geobacter sulfurreducens*, *Shewanella oneidensis* and *Desulfovibrio vulgaris*). Effective concentrations of solvents which inhibited growth rates by 50% (EC_50_) were determined. The octanol-water partition coefficient or log P_o/w_ of a CAH proved a generally satisfactory measure of its toxicity. Most species tolerated approximately 3-fold and 10-fold higher concentrations of the two relatively more polar CAHs CF and 1,2-DCA, respectively, than the two relatively less polar compounds PCE and CT. EC_50_ values correlated well with growth rates observed in solvent-free cultures, with fast-growing organisms displaying higher tolerance levels. Overall, fermentative bacteria were more tolerant to CAHs than respiring species, with iron- and sulfate-reducing bacteria in particular appearing highly sensitive to CAHs. These data extend the current understanding of the impact of CAHs on a range of anaerobic bacteria, which will benefit the field of bioremediation.

Chlorinated aliphatic hydrocarbons (CAHs) contaminate many soil and groundwater sites worldwide. Due to their significant hydrophobicity, resistance to degradation and toxicity, they create long-term sources of pollution and are the target of remediation efforts (8). Bioremediation, a technology relying on bacteria to degrade contaminants, is a proven treatment option for CAH remediation (2, 52). Highly chlorinated CAHs such as perchloroethene (PCE) and carbon tetrachloride (CT) are not susceptible to degradation by aerobic bacteria, but can be detoxified through the action of many types of anaerobic bacteria (21, 38). These include organohalide respiring bacteria (ORB), iron-, sulfate- and nitrate-reducing bacteria, and fermenters (13, 15, 36, 43, 48). These bacteria can either degrade CAHs enzymatically and derive energy in this way or mediate CAH removal cometabolically.

One important limitation of microbially mediated CAH degradation is the occurrence of toxic effects on bacteria. Being lipid-soluble solvents, CAHs partition in bacterial membranes, causing substantial damage to cells when present above certain concentrations (29). The magnitude of this toxicity depends on a solvent’s physicochemical attributes combined with the ability of a particular bacterial species to mount responses to solvent stress. Tolerance mechanisms employed to withstand solvent stress include solvent degradation, modification of membrane and cell wall composition, and active solvent extrusion by efflux pumps (20, 46, 47). Tolerance varies between species, and CAH bioremediation strategies that employ bacteria selected for their superior tolerance to the target solvents are more likely to achieve successful rates and extents of remediation.

However, most knowledge concerning the solvent tolerance of bacteria has been accumulated through studies of aerobic bacteria. For example, it has been shown that the toxicity of a particular solvent is correlated with its hydrophobicity, given by its log P_o/w_ value (28). Log P_o/w_, the logarithm of a solvent’s partitioning in a standard octanol/water two-phase system, represents the ratio of solvent concentrations in each of the two liquids:

$$Log\;P_{o/w}=Log\;\left(\frac{{\left[solvent\right]}_{octanol}}{{\left[solvent\right]}_{water}}\right)$$

High log P_o/w_ solvents are more hydrophobic (or lipophilic) and those with low log P_o/w_ are more hydrophilic. Solvents with log P_o/w_ of between 1 and 4 are thought to be especially toxic to bacteria, as they are water-miscible enough to be bioavailable and lipid-soluble enough to disrupt bacterial membranes and cause cell death (49). Numerous aerobic bacteria able to grow in the presence of free-phase solvents have now been isolated, a large proportion of which belong to the genera *Pseudomonas*, *Bacillus* and *Rhodococcus* (27, 29).

The same level of information is not available concerning anaerobic bacteria. Certain species such as *Clostridium acetobutylicum* and *Zymomonas mobilis* employed in the production of solvents and biofuels have been studied in detail (10, 45); however, very few data exist regarding the solvent tolerance of other anaerobic species important in CAH bioremediation settings. Because CAH-contaminated environments such as soil and aquifers are often complex and inhabited by a variety of species, it is useful to not only understand how solvents influence the species targeted as the catalyst of remediation but also co-existing species which may modulate bioremediation outcomes through mutualism or competition. In this sense, a broad view of the solvent tolerance of different metabolic types of anaerobes is beneficial. While reports of solvent levels tolerated by ORB are available (1, 5, 55), this is not the case for other metabolic types. Duldhardt *et al.* (18, 19) recently determined solvent tolerance levels for the iron reducer *Geobacter sulfurreducens*, the sulfate reducer *Desulfococcus multivorans* and the nitrate reducer *Thauera aromatica* with a number of hydrocarbons relevant to oil contamination; however, no CAHs were tested.

The present article focuses on the sensitivity of anaerobic bacteria to chlorinated solvents. The aim was to generate EC_50_ values for a diverse range of bacteria potentially relevant to CAH bioremediation. The impact of perchloroethene (PCE), carbon tetrachloride (CT), chloroform (CF) and 1,2-dichloroethane (1,2-DCA) on bacterial growth rates was assessed. These four CAHs were chosen as they are classified as priority pollutants (8) and are amenable to degradation by anaerobic bacteria (16, 24, 39, 40).

A broad range of bacteria was chosen based on their environmental origin, relevance to bioremediation, and presence in the existing solvent tolerance literature for ease of data comparison. Of the four fermentative bacteria selected, *Escherichia coli* was chosen due to its widespread use as a model organism for bacteria in general and the large body of literature concerning various aspects of its tolerance to xenobiotic compounds. *Klebsiella* sp. was chosen as a fast-growing fermenter and was isolated from a wastewater treatment plant, which potentially exposed it to a range of compounds and influenced its ability to tolerate solvents. *Clostridium* sp. and *Paenibacillus* sp. were isolated from a CAH-contaminated site and were of particular interest since their long-term exposure to CAHs hypothetically affected their response to solvent stress. Of the four respiratory lineages examined, the nitrate reducer *Pseudomonas aeruginosa* was chosen because of the considerable amount of knowledge regarding the resistance of *Pseudomonas* spp. to organic solvents. The iron reducers *Geobacter sulfurreducens* and *Shewanella oneidensis* were chosen as representatives of ubiquitous respiring anaerobes with bioremediation potential, and finally the sulfate reducer *Desulfovibrio vulgaris* was selected as a representative of sulfate-reducing bacteria, also widespread in anaerobic environments and with potential applications as agents of bioremediation.

## Materials and Methods

### Bacteria and culture conditions

All bacteria included in this study were cultivated under conditions known to favor optimal growth in order to minimize the impact of growth-limiting factors other than CAHs. *Escherichia coli* strain K12 substrain W3110 and *Pseudomonas aeruginosa* strain PAO1 were obtained from the University of New South Wales culture collection. Both species were cultivated under N_2_ in Luria Bertani broth (LB), containing per L: 10.0 g tryptone, 5.0 g yeast extract and 10.0 g NaCl. For *P. aeruginosa*, 2.0 g L^−1^ NaNO_3_ was added as this organism requires NO_3_^−^ as an electron acceptor in the absence of oxygen.

*Klebsiella* sp. strain SM1, *Clostridium* sp. strain BIP1 and *Paenibacillus* sp. strain BIP2 were isolated in our laboratory and deposited in the Belgian Co-ordinated Collection of Microorganism (BCCM) under the respective strain numbers LMG 26928, LMG 26929 and LMG 26926. Isolation and identification procedures are described below. These three bacteria were grown under N_2_ in half-concentrated Brain–Heart Infusion broth (Oxoid, Basingstoke, UK) containing, per L: 8.75 g beef heart infusion solids, 5.0 g proteose peptone, 1.0 g glucose, 2.5 g NaCl and 1.25 g Na_2_HPO_4_ and 0.4 g L^−1^ cysteine-HCl as a reducing agent.

*Geobacter sulfurreducens* strain 12127^T^ and *Desulfovibrio vulgaris* subsp. *vulgaris* strain 2119 were purchased from the German Collection of Microorganisms and Cell Cultures (DSMZ, Braunschweig, Germany). *G. sulfurreducens* was cultivated under N_2_-CO_2_ (80/20 v/v) in the DSMZ-recommended defined medium DSM 826, with 10 mmol L^−1^ acetate as an electron donor and 50 mmol L^−1^ fumarate as an electron acceptor (http://www.dsmz.de, accessed 30/07/2013). *Desulfovibrio vulgaris* was cultivated under N_2_-CO_2_ in DSMZ medium 334, replacing the acetate with 10 mmol L^−1^ lactate as an electron donor and adding 5 mmol L^−1^ sulfate as an electron acceptor. *Shewanella oneidensis* strain MR-1 was purchased from the American Type Culture Collection (ATCC strain 700550) and cultivated under N_2_-CO_2_ in a defined medium described in Löffler *et al.* (37) with 20 mmol L^−1^ lactate as an electron donor and 20 mmol L^−1^ fumarate as an electron acceptor.

### Isolation and identification of fermenting bacteria

Three fermentative bacterial strains were isolated from two environmental sources, namely wastewater and groundwater from a CAH-contaminated site, in order to incorporate bacteria from polluted environments in the study and their response was compared to that of well-studied characterized strains.

Firstly, strain SM1 was isolated from activated sludge from the St Mary’s Wastewater Treatment plant in Sydney, Australia, by spread-plating sludge onto R2A agar plates (Oxoid) and incubating aerobically at 30°C. Secondly, strains BIP1 and BIP2 were isolated either by inoculating groundwater associated with a PCE/CT Dense Non-Aqueous Phase Liquid (DNAPL) sample from a CAH-contaminated site in Sydney, Australia, into defined anaerobic medium containing glucose (BIP1), or by spread-plating onto aerobic LB agar plates (BIP2). For species identification, colony PCRs were performed to amplify the 16S rRNA gene with primers 27F and 1492R (34). Thermal cycling was performed at 95°C for 5 min; this was followed by 30 cycles consisting of 94°C for 1 min, 55°C for 30 s, and 72°C for 1 min. PCR products were purified using silica-membrane-based columns of the QIAquick PCR purification kit (Qiagen, Chadstone, Australia) according to the manufacturer’s instructions and sequenced using an Applied Biosystems 3730 DNA Analyzer. Sequences 600–700 base-pairs in length (both forward and reverse for each strain) were matched against online NCBI nucleotide databases using the Megablast algorithm of the Basic Local Alignment Search Tool (BLAST) (4, 57). Uncultured and environmental sequences were excluded from searches in order to obtain most closely related known organisms.

### Solvent tolerance tests

Solvent tolerance tests were carried out in 25 mL Hungate tubes containing 10 mL medium, capped with Teflon-lined rubber stoppers and sealed with aluminum crimp caps. Incubation temperatures were as follows: *E. coli* 30°C, *P. aeruginosa* 37°C, *S. oneidensis* 30°C, *Klebsiella* sp. 26°C, *Paenibacillus* sp. 30°C, *Clostridium* sp. 24°C, *G. sulfurreducens* 30°C and *D. vulgaris* 30°C. Each species was tested with PCE, CT, CF and 1,2-DCA, each at five different concentrations in triplicate. One set of control triplicates did not include any solvent and served as the benchmark for growth rate comparisons. Each species was tested with all four CAHs at the same time in order to minimize any variations between cultures used on different days.

Solvents were added to autoclaved tubes directly as the free phase or as dilute ethanolic stocks (1 to 100 μL). When ethanolic stocks were used, one set of triplicates was set up including the maximum ethanol concentration (analytical grade ethanol; Ajax Finechem, Sydney, Australia) used in order to control the effect of ethanol on growth. No differences were observed between no-solvent and ethanol-only controls. Tubes were left at room temperature overnight to allow for solvent equilibration between the aqueous phase and headspace before inoculation. Tests were initiated by inoculating exponentially growing parent cultures (5% transfers). Growth was monitored by measuring optical density at 600 nm against medium blanks. Monitoring ceased as soon as the stationary phase was reached, which took between 5 h for fast-growing organisms such as *Klebsiella* sp. and 70 h for slow growers such as *D. vulgaris*.

### Mathematical treatment of data

Actual solvent concentrations in the aqueous phase were calculated using Henry’s constants for each temperature, aqueous and gas phase volumes and volumes of solvent added. For PCE, solvent concentrations above the maximum aqueous solubility of 0.9 mmol L^−1^ are nominal and do not reflect actual dissolved concentrations but rather indicate increasing amounts of free-phase PCE.

Specific growth rates μ (h^−1^) were determined by plotting the natural logarithm of OD_600_ at time “t” (X_t_) over that at time “0” (X_0_) versus time:

$$\mu =\left[ln\;\left(\frac{X_{t}}{X_{0}}\right)\right]÷t$$

Time “0” was taken as the beginning of exponential growth. No differences in lag or exponential phase lengths were observed between no-solvent controls and solvent-containing cultures: bacteria consistently started and ceased growing at the same time and growth only varied in rate. Average specific growth rates were calculated for each set of triplicates and are expressed as percentages of the no-solvent controls’ average growth rate μ_0_ set at 100%. These percentages were plotted against solvent concentrations and the effective concentration causing a 50% decrease in growth rate (EC_50_) was estimated by linear interpolation and in a few cases by extrapolation. Growth rates of zero were assigned to cultures where OD_600_ readings either decreased or did not increase above 0.04 during tests.

The fate of CAHs in bacterial cultures was checked by gas chromatography and flame ionization detection (GC-FID) as described elsewhere (33). The CAHs were never utilized as carbon sources by the bacteria and remained stable.

## Results

This study included the isolation and identification of three fermentative bacterial strains from environmental sources. Following 30°C incubation of R2A agar plates on which 10 μl activated sludge had been spread, a single colony was picked and inoculated into liquid aerobic LB medium. The purity of this isolate, named SM1, was confirmed by microscopic examination and by spread-plating. Partial sequences of strain SM1’s 16S rRNA gene (DDBJ accession number AB858357) matched the 16S rRNA gene sequences of various strains of *Klebsiella oxytoca* with 100% similarity; hence, this bacterium is referred to as *Klebsiella* sp. strain SM1.

Following the inoculation of CAH-contaminated groundwater into anaerobic glucose medium, growth at 30°C was observed by increased turbidity and acid formation within one month. Small white colonies appeared in R2A agar roll tubes inoculated with this glucose-fermenting culture. Colonies were picked and inoculated into anaerobic liquid R2A medium. Culture purity was confirmed microscopically and by spreading onto R2A and LB agar plates, incubated both aerobically and in an anaerobic jar at 30°C. The isolate, named BIP1, could not grow aerobically and formed large white colonies on anaerobic R2A agar plates only. It was identified as a member of the *Clostridium* genus by partial 16S rRNA gene analysis (DDJB accession number AB858358), most closely matching *Clostridium* sp. strain BL-26 (99.8% similarity, GenBank accession number DQ196630.2).

The same groundwater collected above a CAH-DNAPL layer was spread onto LB plates which were incubated aerobically at 30°C. A single colony was picked and re-streaked, then inoculated into both aerobic and anaerobic liquid LB medium. This isolate, named strain BIP2, was able to grow in both conditions and was characterized as a strain of *Paenibacillus stellifer*, its partial 16S rRNA gene sequence (DDJB accession number AB858359) most closely matching *Paenibacillus stellifer* strain Gt48 (99% similarity, GenBank accession number GU321993.1). It is referred to hereafter as *Paenibacillus* sp. strain BIP2.

**Fig. 1:**
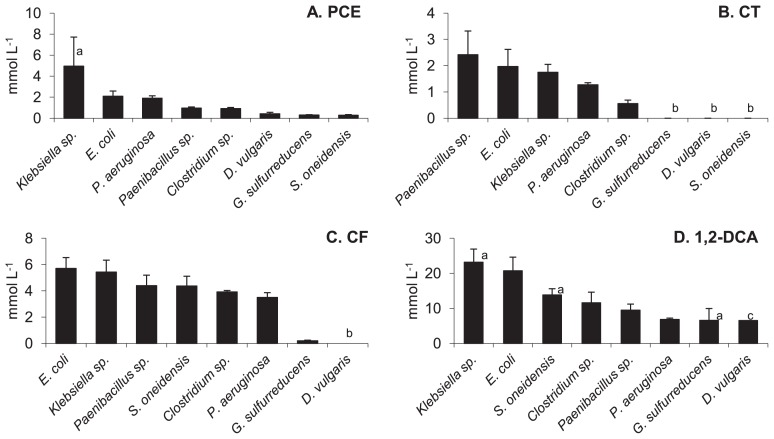
Concentrations of chlorinated solvents which inhibit growth rates by 50% (EC_50_). PCE concentrations above 0.9 mmol L^−1^ are nominal and indicate the presence of free-phase PCE. Values are averages of triplicates; error bars represent one standard deviation. ^a^Value obtained by extrapolation using linear trend line; ^b^No growth occurred at the lowest solvent level tested; ^c^*Desulfovibrio vulgaris* tolerated far above the highest level of 1,2-DCA tested (4.65 mmol L^−1^) and was assigned the same EC_50_ as *Geobacter sulfurreducens*.

All eight anaerobic bacteria tested were negatively affected by the presence of CAHs in the growth medium, and solvent concentrations found to decrease their growth rates by half are reported in Fig. 1. All eight species were able to grow at 50% of no-solvent rates (μ_0_) with 0.3 mmol L^−1^ PCE or more (Fig. 1A). *Klebsiella* sp. was the species most tolerant to PCE, and could still grow at 54% of μ_0_ at the maximum nominal concentration tested of 4.58 mmol L^−1^; hence, its EC_50_ value for PCE was estimated by extrapolation at 4.95 mmol L^−1^. Except for *S. oneidensis*, *D. vulgaris* and *G. sulfurreducens*, all bacteria had EC_50_ values for PCE above this solvent’s maximum solubility of 0.9 mmol L^−1^ which means they were still able to grow at or above 50% of μ_0_ when PCE was present as a separate phase. Further increases in nominal PCE concentrations led to eventual complete inhibition of growth, indicating that free-phase PCE itself exerted toxic effects. *S. oneidensis*, *D. vulgaris* and *G. sulfurreducens* were affected by PCE below the point of free-phase appearance.

With CT (Fig. 1B), *S. oneidensis*, *D. vulgaris* and *G. sulfurreducens* were unable to grow even at the lowest levels tested of 80, 80 and 30 μM, respectively. The other five species displayed EC_50_ values between 0.5 and 2.4 mmol L^−1^, corresponding to between 10 and 50% of CT’s aqueous solubility of 5 mmol L^−1^. *Paenibacillus* sp., isolated from a CAH-contaminated site, was the organism most tolerant to CT with an EC_50_ value of 2.4 mmol L^−1^.

In general, the anaerobic bacteria under study could tolerate more CF and 1,2-DCA than PCE and CT (Fig. 1C and 1D). With the exception of *G. sulfurreducens* and *D. vulgaris*, all microorganisms displayed EC_50_ values for CF above 3.5 mmol L^−1^. *G. sulfurreducens* showed an EC_50_ for CF of 0.2 mmol L^−1^, while *D. vulgaris* was the organism most sensitive to CF and was completely inhibited even at the lowest test concentration of 0.2 mmol L^−1^. All eight lineages could tolerate more 1,2-DCA in the medium than any other solvent, EC_50_ values being consistently above 6.5 mmol L^−1^ (Fig. 1D). *G. sulfurreducens* and *D. vulgaris* could still grow at 70% and 91% of μ_0_ at the respective highest 1,2-DCA levels tested of 3.72 and 4.65 mmol L^−1^, and while an EC_50_ of 6.5 mmol L^−1^ could be calculated by extrapolation for *G. sulfurreducens*, it could not be calculated for *D. vulgaris* given the lack of a decreasing trend of growth rate versus 1,2-DCA. Because *D. vulgaris* was at least as tolerant to 1,2-DCA as *G. sulfurreducens*, a minimum EC_50_ of 6.5 mmol L^−1^ is reported for this species. Overall, the most solvent-tolerant anaerobic bacteria were *Klebsiella* sp. and *E. coli*, consistently appearing in the top three highest EC_50_ values for any of the four solvents tested. The least tolerant organisms were *D. vulgaris* and *G. sulfurreducens*.

**Fig. 2:**
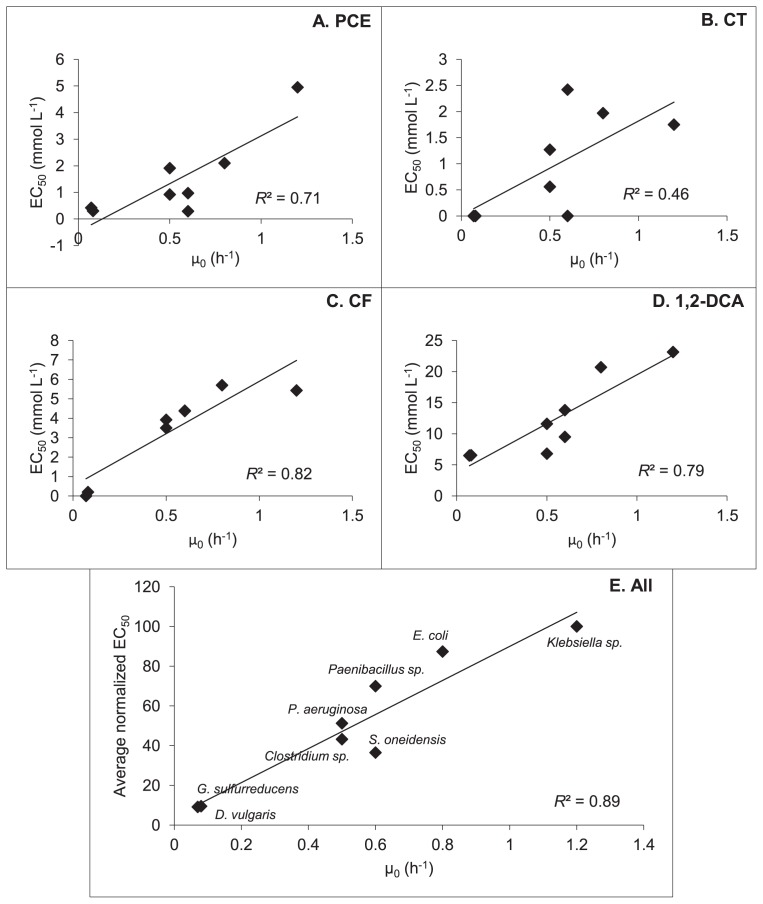
Relationship between no-solvent growth rate (μ_0_) and EC_50_. Panel E comprises a plot of species’ averaged normalized EC_50_s versus no-solvent growth rates. Normalization was carried out by setting the EC_50_ of *Klebsiella* sp. at 100% for each solvent and expressing EC_50_s for all other species as percentages of *Klebsiella*’s EC_50_. All four values for each species were then averaged.

The ability of a species to tolerate CAHs, as given by EC_50_, correlated well with its growth rate in solvent-free cultures, μ_0_ (Fig. 2). Coefficients of determination for linear regressions fitted to the data were above 0.7 for all solvents except CT (Fig. 2B), validating growth rate as an important parameter influencing bacterial solvent tolerance. In the case of CT, the ability of a species to grow rapidly under stress-free conditions did not influence its capacity to tolerate the chlorinated compound, as it did with the other three CAHs. Nevertheless, when scoring a species’ solvent tolerance by normalizing and averaging its EC_50_ values across all four CAHs, a very good correlation appears between the maximum growth rate and tolerance (*R*^2^ = 0.89, Fig. 2E). Slow-growing organisms such as *G. sulfurreducens* and *D. vulgaris* were the most sensitive overall, while fast-growing species such as *Klebsiella* sp. and *E. coli* demonstrated marked resistance to CAH toxicity.

**Fig. 3:**
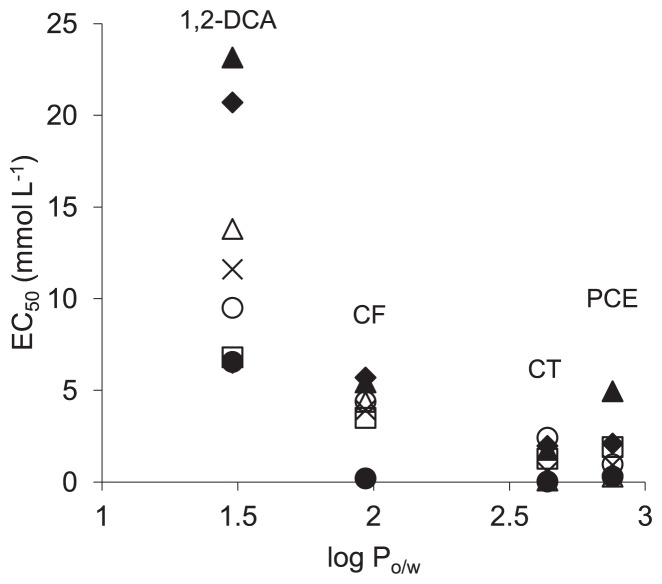
Relationship between EC_50_ and CAH log P_o/w_ (dimensionless) for each species. Log P_o/w_ values were obtained from (35): 1,2-DCA: 1.48; CF: 1.97; CT: 2.64; PCE: 2.88. Legend: ♦ *E. coli*; □ *P. aeruginosa*; ▲ *Klebsiella* sp.; × *Clostridium* sp.; ○ *Paenibacillus* sp.; ● *G. sulfurreducens*; ⋄ *D. vulgaris*; △ *S. oneidensis*.

When considering individual CAHs, a good correlation could be found between a compound’s hydrophobicity, as denoted by its log P_o/w_, and its ability to inhibit bacterial growth (Fig. 3). For all eight bacterial lineages tested, 1,2-DCA (log P_o/w_ = 1.48) was consistently less toxic than CF (log P_o/w_ = 1.97), CT (log P_o/w_ = 2.64) and PCE (log P_o/w_ = 2.88). For all but two species, CF was less toxic than CT and PCE, the exceptions to this rule being the two respiring species *G. sulfurreducens* and *D. vulgaris*. The delineation between CT and PCE was less clear. Except for *Paenibacillus* sp. and despite a marginally lower log P_o/w_, CT was consistently more toxic than PCE, as it took less CT to diminish growth rates by half than PCE. Of particular note is the severe impact of CT on the three respiring strains *S. oneidensis*, *G. sulfurreducens* and *D. vulgaris*, which showed no growth at all at or below 80 μM.

## Discussion

The rapid and complete bioremediation of CAHs in soil and groundwater hinges on the capacity of the bacteria involved to tolerate high concentrations of these solvents. Depending on the solvents present and on the cleanup protocol to be implemented, one or several anaerobic genera can be selected as detoxifying agents. Very few reports concerning the solvent tolerance of anaerobic bacteria are available, which represents a gap in the bioremediation knowledge base. In this report, the CAH tolerance of fermenting, iron-, sulfate-, nitrate-and fumarate-reducing bacterial species was studied.

Studies on the sensitivity of aerobic bacteria to solvents have led to an empirical rule concerning solvent toxicity, stating that a bacterium can grow with a free phase of any solvent with a log P_o/w_ above that of the index value, equivalent to the log P_o/w_ of the most toxic solvent tolerated (6, 7, 28). Solvents with log P_o/w_ below the index value are too toxic to allow growth when present as a free phase.

Results of the current study support the validity of this rule for anaerobic bacteria. The CAH with the highest log P_o/w_ was PCE, and five out of eight species tolerated the presence of a small volume of free-phase PCE, indicated by EC_50_ values above the solubility of PCE (0.9 mmol L^−1^). No species could tolerate the presence of free phases of any of the other three CAHs with lower log P_o/w_. While EC_50_ values for the more polar CF and 1,2-DCA were higher than for the less polar CT and PCE, these EC_50_ values actually represent smaller percentages of the solvents’ maximum aqueous solubility. The average EC_50_ for CT was 1 mmol L^−1^, equivalent to 20% of CT solubility, while average EC_50_s for CF and 1,2-DCA were 3.5 and 12.3 mmol L^−1^, equating to 5 and 14% of maximum solubility respectively. This indicates that, overall, the anaerobic bacteria tested would be less able to grow with free phases of more polar than less polar compounds. This has implications for the bioremediation of solvents present as DNAPL, suggesting that more polar solvents such as 1,2-DCA are less likely to be tolerated as free phases than as dissolved plumes. With less polar compounds such as PCE, the likelihood of bacteria establishing themselves near DNAPL is higher, leading to faster dissolution times as degradation proceeds (12).

The increased toxicity of PCE to bacteria beyond its aqueous solubility threshold observed here reflects phenomena noted by other authors (9, 56). One potential explanation involves direct contact between cells and free-phase solvent increasing as the number and size of solvent droplets increased (56), thereby amplifying solvent partitioning in membranes and causing cell death.

Despite CT having a log P_o/w_ similar to that of PCE, it was more toxic than PCE for most species examined. The higher aqueous solubility limit of CT possibly accounts for this divergence. While PCE forms a free phase above 0.9 mmol L^−1^, CT’s aqueous solubility is 5 mmol L^−1^, meaning greater dissolved levels of CT can cause stress to bacterial cells before a separate phase is formed. Between 0.9 and 5 mmol L^−1^, where many EC_50_s are found, CT presents a much greater threat to cell integrity compared with PCE, by still existing in the dissolved form while being almost as hydrophobic as PCE.

In terms of solvent tolerance, the eight anaerobes investigated here can be classified into three groups. Firstly, the two fermenters *E. coli* and *Klebsiella* sp. displayed the highest resistance to CAH stress. They belong to the family Enterobacteriaceae of the class *Gammaproteobacteria*, are facultatively anaerobic, and were also the fastest growing bacteria tested. In the second category are the other two fermenting species *Clostridium* sp. and *Paenibacillus* sp., both belonging to the phylum *Firmicutes*, together with the two other *Gammaproteobacteria P. aeruginosa* and *S. oneidensis*. These four species displayed intermediate growth rates and EC_50_ values. In the third category are the two *Deltaproteobacteria G. sulfurreducens* and *D. vulgaris*, the most CAH-sensitive species in this investigation and also the slowest growing and only strictly anaerobic respiring species.

When comparing EC_50_ values obtained in this study with those given in the literature for aerobic bacteria, tolerance levels appear similar between aerobes and the fast-growing anaerobes of the first two groups outlined above. In a study by Heipiepier *et al.* (26), EC_50_s of 3.6 and 3.2 mmol L^−1^ were reported for aerobic *Pseudomonas putida* exposed to toluene and styrene, two solvents with log P_o/w_ of 2.48 and 3.0, respectively. Hage *et al.* reported EC_50_s of 8.0, 13.0, 1.5, 3.0, 0.5 and 1.9 mmol L^−1^ for *P. putida* growing in the presence of various organic compounds with log P_o/w_ in the range 1.72–3.46 (25). Another aerobe, *Acinetobacter calcoaceticus*, showed EC_50_s of between 6.0 and 0.11 mmol L^−1^ when exposed to alkanols of log P_o/w_ of between 1.87 and 3.97, with EC_50_s decreasing with increasing log P_o/w_ (30). In this study, most bacteria tested showed EC_50_s >0.5–23 mmol L^−1^ with solvents of log P_o/w_ of 1.48–2.88. Hence, we conclude that anaerobic bacteria can be at least as tolerant as aerobic bacteria, depending on metabolism and growth rate.

The apparent relationship between growth rate and CAH tolerance could be explained by a high turnover of cell components in fast-growing species compensating for the damage inflicted through contact with CAHs. Further, a number of solvent tolerance mechanisms elucidated in Gram-negative bacteria, such as changes in membrane fatty acid composition and production of stress proteins, require *de novo* synthesis, strongly linked to cell growth (30, 32, 46). Gram-negative bacteria have also been shown to possess energy-dependent solvent efflux pumps (31, 54), pointing to energy status as an important factor in solvent resistance. In Gram-positive bacteria, changes in membrane composition and the appearance of stress proteins have also been demonstrated (3, 51). The sensitivity of slow-growing organisms in this study is likely linked to lower energy generation rates, leading to a delay in resistance mechanism activation and thus greater vulnerability to solvent attack. Duldhardt *et al.* (19) recently demonstrated that the anaerobic respirers *G. sulfurreducens*, *Desulfococcus multivorans* and *Thauera aromatica* respond to solvent toxicity through increases in the saturated fatty acid component of their cell membranes, a mechanism dependent on cell growth.

The compound CT was particularly toxic to *G. sulfurreducens*, *D. vulgaris* and *S. oneidensis*. When comparing EC_50_ values obtained for *G. sulfurreducens* and *D. multivorans* in a study by Duldhardt *et al.* (18) to those obtained here for *G. sulfurreducens* and *D. vulgaris*, close similarities are found for chlorinated and non-chlorinated solvents of similar log P_o/w_. For instance, EC_50_s for 1,2-DCA of 6.5 mmol L^−1^ obtained here approximate the EC_50_s of 7.6 to 8.9 mmol L^−1^ obtained by Duldhardt *et al.* for phenol, which has the same log P_o/w_ as 1,2-DCA (log P_o/w_ = 1.48). Also, EC_50_s of 0.3–0.4 mmol L^−1^ were determined here for PCE (log P_o/w_ = 2.88) compared with EC_50_s of 0.1–0.2 mmol L^−1^ for solvents of log P_o/w_ near 3.0, such as 1-octanol and ethylbenzene, found by Duldhardt *et al.* The same correspondence, however, did not apply to CT and CF and non-chlorinated compounds with similar log P_o/w_ values, suggesting that CT and CF have additional distinct mechanisms of toxicity. A specific toxicity mechanism has previously been reported for CT, with the formation of radicals from CT interacting with reduced bacterial proteins and co-factors (14, 42). This is termed “reactive toxicity” and solvents which cause this type of toxicity do not fit models based on properties such as log P_o/w_ (9, 44). It is possible that electron chain components located in the cell membrane of respiring bacteria are more vulnerable to this toxic action than cytoplasm-based energy generation through fermentation. The toxicity of CT and CF to organohalide-respiring bacteria such as *Dehalococcoides* is well-documented (17, 41), suggesting the general susceptibility of anaerobic respiring bacteria to these compounds.

The nitrate respirer *P. aeruginosa*, despite possessing an electron chain, was not more susceptible to CT or CF than other solvents. Its high growth rate coupled with the well-known ability of *Pseudomonas* species to tolerate organic solvents in general (46) likely account for this exception. Moreover, the sensitivity of electron transport chain proteins to solvents might differ between nitrate respiration and other respiration pathways and is worthy of further research efforts.

Of note are the two fermenting bacteria *Clostridium* sp. strain BIP1 and *Paenibacillus* sp. strain BIP2, both isolated from a CAH-contaminated aquifer as part of this study. Several members of the Clostridia have been found to be associated with organochlorine-respiring bacteria (ORB) in dechlorinating cultures, where they are thought to participate in substrate fermentation and hydrogen supply to the ORB (22, 23, 50). Bowman *et al.* (11) tested the effects of 1,2-DCA and PCE on hydrogen production by 18 strains of *Clostridium* isolated from CAH-contaminated groundwater, and inhibitory concentrations fit well with EC_50_ values obtained here for *Clostridium* sp. strain BIP1. Interestingly, the 16S rRNA gene of strain BIP1 most closely matched *Clostridium* sp. strain BL-26 isolated by Bowman *et al.*, and it was also isolated from acidic (pH 3.5 to 5.8) CAH-contaminated groundwater, suggesting that similar strains of *Clostridium* sp. may exist in acidic chlorinated solvent-polluted groundwater sites around the world. The *Paenibacillus* sp. strain examined here was isolated from the same contaminated site and was the most CT-tolerant bacterium amongst all those tested. It is possible that long-term *in situ* exposure to DNAPL comprising CT led to the increased tolerance of this organism. The potential of these groundwater-dwelling bacteria to act as hydrogen providers, together with their relatively high CAH tolerance compared with respiring species, suggests that encouraging the co-existence of fermenting and respiring species may have advantages over the stimulation of respiring species only in bioremediation strategies.

In summary, the data presented indicate that anaerobic bacteria are sensitive to CAHs, and their level of toxicity can generally be predicted by their log P_o/w_. However, log P_o/w_ is not always a reliable indicator and other parameters such as aqueous solubility and potential for reactive toxicity need to be evaluated when assessing the impact of CAH contamination in an aquifer or planning a bioremediation strategy. Fast-growing bacteria, especially fermenters, were found to be more tolerant overall than slow-growing anaerobic respiring bacteria, likely due to their capacity to rapidly activate energy-dependent resistance mechanisms. Efforts are underway in our laboratory to further elucidate possible links between growth rate and solvent tolerance. Bioremediation protocols would benefit from including fermenting organisms in their CAH detoxification plan, as not only can they often degrade contaminants through cometabolism but also new ways of harvesting the reducing power they liberate from organic carbon are continuously being discovered (53). Moreover, fermenting bacteria can provide substrates and co-factors to organochlorine-, sulfate- and iron-reducing bacteria, which affect CAH dechlorination directly through enzymatic action or through the production of redox-active Fe(II) and sulfide minerals. The higher sensitivity of IRB and SRB shown here must be considered when these organisms are chosen as biocatalysts: the location of Fe(II) and/or sulfide-rich zones at contaminated sites for CAH treatment is likely to be most successful down-gradient of DNAPL zones, where solvent concentrations can be tolerated without compromising the rate or extent of treatment. Whether fermenting bacteria can enhance this process is worthy of further research.
